# Innate Immune Memory in Monocytes and Macrophages: The Potential Therapeutic Strategies for Atherosclerosis

**DOI:** 10.3390/cells11244072

**Published:** 2022-12-15

**Authors:** Zhigang Guo, Lixue Wang, Hongjian Liu, Yuhuai Xie

**Affiliations:** 1 Huanghe Science and Technology College, Zhengzhou 450006, China; 2 Shandong Provincial Key Laboratory of Animal Biotechnology and Disease Control and Prevention, College of Animal Science and Technology, Shandong Agricultural University, Tai’an 271018, China; 3 Department of Pharmacy, The Second Affiliated Hospital of Shandong First Medical University, Tai’an 271000, China; 4 Department of Immunology, School of Basic Medical Sciences, Fudan University, Shanghai 200032, China

**Keywords:** atherosclerosis, innate immune memory, metabolic reprogramming, epigenetic rewiring, macrophage

## Abstract

Atherosclerosis is a complex metabolic disease characterized by the dysfunction of lipid metabolism and chronic inflammation in the intimal space of the vessel. As the most abundant innate immune cells, monocyte-derived macrophages play a pivotal role in the inflammatory response, cholesterol metabolism, and foam cell formation. In recent decades, it has been demonstrated that monocytes and macrophages can establish innate immune memory (also termed trained immunity) via endogenous and exogenous atherogenic stimuli and exhibit a long-lasting proinflammatory phenotype. The important cellular metabolism processes, including glycolysis, oxidative phosphorylation (OXPHOS), the tricarboxylic acid (TCA) cycle, fatty acid synthesis, and cholesterol synthesis, are reprogrammed. Trained monocytes/macrophages with innate immune memory can be persistently hyperactivated and can undergo extensive epigenetic rewiring, which contributes to the pathophysiological development of atherosclerosis via increased proinflammatory cytokine production and lipid accumulation. Here, we provide an overview of the regulation of cellular metabolic processes and epigenetic modifications of innate immune memory in monocytes/macrophages as well as the potential endogenous and exogenous stimulations involved in the progression of atherosclerosis that have been reported recently. These elucidations might be beneficial for further understanding innate immune memory and the development of therapeutic strategies for inflammatory diseases and atherosclerosis.

## 1. Introduction

Atherosclerosis is a degenerative disorder of the large- and medium-sized arterial walls characterized by chronic low-grade inflammation and the accumulation of apoB-containing lipoproteins [1]. These events result in the persistent recruitment of monocyte-derived macrophages from the circulatory system, which become the most abundant innate immune cells of the plaques in the various pathophysiological stages of atherosclerosis [2,3]. Monocyte-derived macrophages are the primary cells responsible for inflammation and the major drivers of atherosclerosis initiation and progression. For a long time, it was thought that immunological memory was the specific characteristic of adaptive immunity. Immunological memory is defined as the ability of immune cells to specifically “remember” the first encounter with a specific antigen and trigger a faster and greater secondary response compared to the primary response. Naïve T and B cells have the capacity to develop into antigen-specific and long-lasting memory cells, providing specific and effective elimination and protection against reinfection after the first encounter with a pathogen [4]. However, in recent decades, a growing body of evidence in human and mouse models has revealed that the innate immune system is also able to build long-term memory and acquire enhanced response capabilities towards certain stimuli. This immune response phenomenon is termed innate immune memory or trained immunity [5] and is based on the rewiring of intracellular metabolic processes and epigenetic reprogramming in the cells of the innate immune system, such as monocytes, macrophages, and NK cells, following stimulation with microbial products or endogenous sterile compounds [6,7].

Monocytes and macrophages have been shown to exhibit long-term proinflammatory and proatherogenic memory with an enhanced cytokine response upon brief exposure to inflammatory stimulation, such as Toll-like receptor agonists, which have been proven to be important factors for the development of atherosclerosis [8]. Importantly, endogenous stimulations that are known to be closely associated with atherosclerosis, such as lipoprotein, oxLDL, and aldosterone, can also induce innate immune memory, contributing to enhanced proinflammatory cytokine production and increased foam cell formation [7,9,10]. These phenotypical alterations that occur in monocytes and macrophages with well-established innate immune memory are orchestrated through epigenetic modifications (e.g., DNA methylation, histone methylation, and acetylation) and metabolic regulation that involve a number of central cellular metabolic pathways, including the fatty acid metabolism, cholesterol synthesis, glycolysis, TCA, OXPHOS, and amino acid metabolism pathways [11,12,13,14]. In this review, we focus on the most relevant and recent reported advances in the mechanisms of the monocyte immune-metabolic reprogramming and epigenetic rewiring associated with atherosclerosis as well as the important stimulations reported in monocyte/macrophage innate immune memory during the development of atherosclerosis.

## 2. The Mechanisms of Innate Immune Memory Formation

The induction of innate immune memory is mediated by highly integrated signaling, epigenetic, and metabolic events. The first exposure to a dangerous stimulus results in a functional immune status alteration of innate immune cells. This immune activation status regresses to the basal level but primes the prolonged epigenetic reprograming when abolishing the stimulus. However, both gene expression and cellular immune responses are ferociously enhanced at a much higher level when re-exposed to similar challenges [15]. Meanwhile, during this process, innate immune cells reprogram metabolism progress to accommodate the enhanced energy requirements and provide basic building blocks for the establishment of innate immune memory. Chromatin architectures are modified to initiate or suppress gene transcription, supporting an immediate response when exposed to a similar stimulation. For instance, a number of inflammatory genes are modulated from the repressed state to the activated state by positive histone modifications at the promoter or enhancer sites or the gene body. Importantly, innate immune memory can promote the expansion and differentiation of bone marrow progenitor cells, therefore accelerating the activation of innate immune cells with a trained phenotype, which represents a potent proinflammatory response to a secondary stimulation [11].

**Figure 1:**
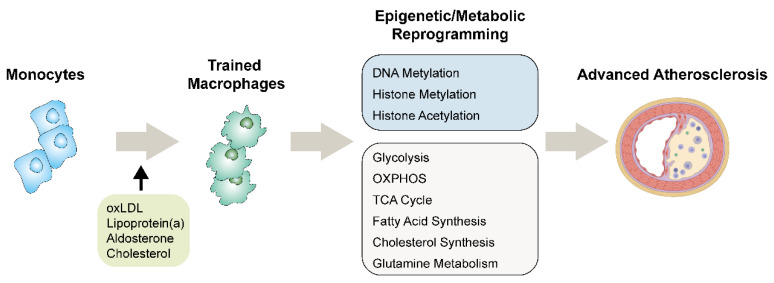
The functions and mechanisms of activated innate immune memory in monocytes/macrophages during the development of atherosclerosis. The activated innate immune memory of macrophages induces epigenetic and metabolic reprogramming, which increases the production of proinflammatory cytokines, leading to a proatherogenic phenotype.

Atherosclerosis is a prolonged arterial inflammatory disease that is involved in many vital functions of monocytes and macrophages. The most widely studied are the inflammatory functions of M1 and M2 macrophages, which are associated with the innate immune memory induced epigenetic and metabolic rewiring [16]. Studies in recent decades revealed that the distinct functions of these two types of macrophages undergo different metabolic processes and perform different functions. Driven by diverse proatherogenic stimuli, both macrophages in the atherosclerotic lesions and monocytes from the circulation can rewire their cellular metabolisms and reprogram their chromatin structures to accommodate the proinflammatory and proatherogenic states. During these processes, the intermediate metabolites derived from various intracellular metabolic pathways also serve as important functional molecules in distinct ways. One metabolite can activate and participate in different metabolic processes of a cell. A classic example is the different functions of arginine in LPS/IFN-γ-activated proinflammatory M1 macrophages and IL-4-induced anti-inflammatory M2 macrophages [17]. Glucose also shows divergent usage. It is primarily metabolized via glycolysis in M1 macrophages, and it is primarily metabolized via OXPHOS in M2 macrophages. These different glucose metabolism pathways result in different amounts and production rates of ATP to support cell growth, division, and responses to the environment. Under specific cellular statuses or environments, innate immune cells tend to modulate or transform metabolic pathways to satisfy intracellular functional desires [18,19]. Evidence has proven that monocytes and macrophages subject to innate immune memory exhibit alterations in their metabolic pathways and diverse usages of metabolites [6,11]. Moreover, these trained monocytes/macrophages always show proinflammatory features and acquire a proatherogenic phenotype (Figure 1) [20].

Recent studies have indicated that monocytes/macrophages with innate immune memory are present in patients with atherosclerosis or related risk factors. Circulating monocytes from patients with severe atherosclerosis exhibited a potent ex vivo proinflammatory chemokine and cytokine response upon LPS stimulation compared with healthy subjects without atherosclerosis [12]. Histone modifications at the promoters of proinflammatory genes can be altered in trained monocytes and play an important role in the development of atherosclerosis. Studies have also revealed that metabolic reprogramming is carried out in trained macrophages. All these alterations induce the proinflammatory and proatherogenic phenotypes of monocytes and macrophages. Although some studies have been conducted, more research needs to be carried out to identify potential therapeutic approaches for atherosclerosis based on the mechanisms of innate immune memory and epigenetic rewiring.

## 3. Innate Immune Memory Induced Cellular Metabolism Reprogramming Is Involved in Atherosclerosis

Cellular metabolism alterations have been revealed to be important determinants of immunological responses in innate immune cells. Monocytes and macrophages undergo wide metabolic rewiring (e.g., glycolysis, oxidative phosphorylation, fatty acids synthesis, and amino acid synthesis) and epigenetic reprogramming (e.g., histone modification) during the establishment of innate immune memory to immediately respond to external restimulation. An understanding of the metabolic reprogramming and epigenetic rewiring induced following infection or stimulation can help us to develop new therapies for inflammatory diseases, such as atherosclerosis.

### 3.1. Glycolysis and Oxidative Phosphorylation 

Macrophages show a plasticity feature and undergo two different polarization states, i.e., the classically proinflammatory M1 phenotype and the alternatively activated anti-inflammatory M2 phenotype [21,22]. In disease states, chronic inflammation can induce the aberrant reprogramming of macrophage responses, which leads to a shift in their phenotypes. For instance, this alteration contributes to the increased proinflammatory macrophages in diabetes mellitus and the enhanced anti-inflammatory macrophages during cancer [23]. In atherosclerotic plaques, M1 macrophages have been shown to be enriched and to secrete proatherosclerotic cytokines that are distinct from M2 macrophages and facilitate tissue repair and homeostasis [24]. Interestingly, trained macrophages largely undergo increased glucose consumption and glycolytic flux via lactate and exhibit impaired mitochondrial oxidative phosphorylation, which contribute to their polarization to proinflammatory M1 macrophages [25,26]. 

**Figure 2:**
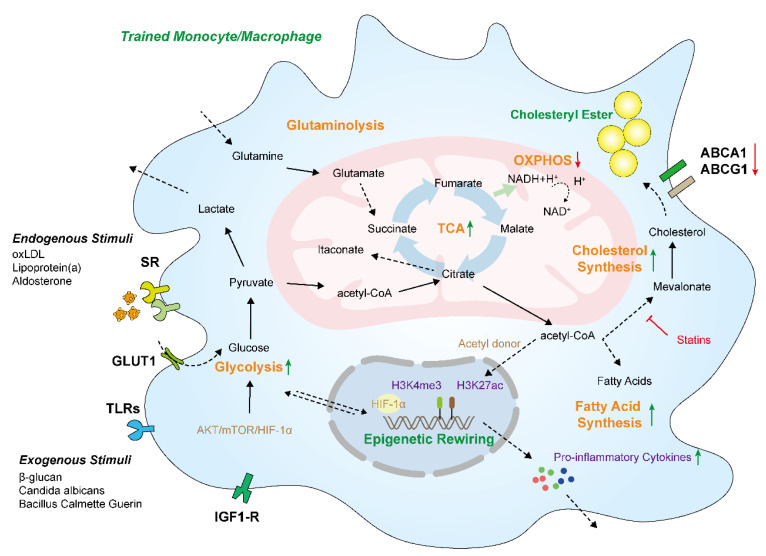
Schematic overview of metabolic and epigenetic reprogramming of monocytes/macrophages with innate immune memory during atherosclerosis. Monocytes/macrophages trained with endogenous or exogenous atherogenic stimuli induce the alteration of important metabolic processes, including glycolysis, oxidative phosphorylation (OXPHOS), the tricarboxylic acid (TCA) cycle, fatty acid synthesis, cholesterol synthesis, and glutaminolysis. These processes subsequently contribute to epigenetic rewiring (histone modifications) at the promoters of a series of genes. Trained monocytes/macrophages exhibit a proinflammatory phenotype by increasing the production of proinflammatory cytokines. IGF1-R, insulin-like growth factor 1 receptors; TLR, Toll-like receptor; SR, scavenger receptor; GLUT1, glucose transporter type 1; ABCA1, ATP-binding cassette transporter A1; ABCG1, ATP-binding cassette transporter G1.

Generally, glucose breakdown can quickly produce ATP and pyruvate. Then, pyruvate is used for the TCA cycle or is further oxidized to lactate [27,28]. Accordingly, glycolysis is promoted in trained and proinflammatory macrophages. During this process, high levels of glucose are consumed, and more glycolysis end-product lactate is released from the cell rather than entering the mitochondria for oxidation [29]. The alteration of this metabolism pathway is mediated by the Akt/mTOR/HIF-1α (hypoxia-inducible factor-1α) signaling pathway, which is important for the effective establishment of innate immune memory [11,25]. The cells in most atherosclerotic plaques undergo fast proliferation and activated cellular metabolism, resulting in limited available oxygen for cells and enriched hypoxia regions. When macrophages are exposed to low oxygen, HIF-1α can be highly expressed and subsequently triggers and stabilizes glycolytic metabolism by upregulating key glycolytic-flux-associated proteins, such as glucose transporter type 1 (GLUT1), 6-phosphofructo-2-kinase/fructose-2,6-bisphosphatase (PFKFB3), and hexokinase II (HK-II). More glucose is taken by the cells and used for glycolysis. It is metabolized to lactate. Meanwhile, the OXPHOS pathway is reduced during this process (Figure 2) [30]. The large quantities of glycose taken up by these activated macrophages lead to high levels of cytokine production via HIF-1α. Although glycolysis is not an efficient ATP production pathway (only two ATP molecules are generated per glucose molecule), it can be rapidly triggered and amplified to produce ATP for cellular activation and responses to the environment [31]. The enhanced glycolytic rate in trained monocytes can be promoted by increased levels of rate-limiting glycolysis enzymes via the increased histone H3K4 trimethylation (H3K4me3) and decreased H3K9 trimethylation (H3K9me3) levels induced by β-glucan [32,33]. Although glycolysis is important for boosting the inflammation that drives the development of atherosclerosis, it seems that increased glycolysis in macrophages alone is insufficient to trigger atherosclerosis due to the fact that no alteration of inflammatory gene expression was observed in GLUT1-overexpressed mice and a J774 macrophage cell line [34].

Compared with glycolysis, oxidative respiration can produce more ATP (36 molecules of ATP are generated per glucose molecule) via the TCA cycle and mitochondrial machinery but at a slower rate compared with glycolysis [35]. In OXPHOS, the electron donor nicotinamide adenine dinucleotide (NADH) and flavin adenine dinucleotide (FADH_2_) are oxidized and provide electrons to the electron transport chain, enabling the respiratory chain to function. In mitochondria, these electrons sequentially transfer through electron transport chain (ETC) complex I or II and consume oxygen to pump protons across the inner membrane, resulting in water generation and a proton gradient. There, the shuttled protons actuate the synthesis of ATP by ATP synthase [36]. However, the balance between glycolysis and OXPHOS is disturbed in trained innate immune cells. It has been reported that β-glucan-induced innate immune memory tends to increase glycolysis and decrease OXPHOS [25], while oxLDL-induced innate immune memory promotes both the glycolytic and OXPHOS pathways [13]. Researchers found that decreased electrons in the OXPHOS pathway might result in a higher NADP+/NADPH ratio in trained cells, which may affect mitochondrial metabolism, while an oxLDL-induced monocyte shows high mitochondrial ROS production, which can be decreased by mTOR inhibition [37]. However, the glycolysis process is always promoted in trained innate immune cells. Therefore, the repression of glycolysis might help to inhibit the enhanced production of proinflammatory cytokines, which could be a potential approach for the treatment of inflammation-induced diseases, such as atherosclerosis. 

### 3.2. Tricarboxylic Acid Cycle

Tricarboxylic acid cycle (TCA cycle, also known as the Krebs cycle) reprogramming is a common characteristic of trained innate monocytes. Macrophages with well-built innate immune memory show an enhanced flux of glucose via the TCA cycle and an increased accumulation of TCA cycle intermediates, such as succinate, citrate, and itaconate [38,39]. The TCA cycle can be disrupted after citrate and succinate sites in trained macrophages. Citrate can be popped for fatty acid biosynthesis, resulting in the enhanced production of inflammatory prostaglandins (Figure 2) [40]. In addition, the withdrawal of citrate is also related to the enhanced itaconate production. It has also been proven that citrate can be transported out of the mitochondria to convert to acetyl CoA as an acetyl donor. Importantly, trained monocytes acquire enhanced histone acetylation in proinflammatory genes to increase their accessibility to transcription factors upon secondary stimulation. Therefore, the enhanced extramitochondrial acetyl CoA from the TCA cycle can serve as a primary acetyl group donor, which may result in histone acetylation in trained macrophages [41]. Itaconate is a metabolite of the TCA cycle and has been proven to be an important contributor to innate immunity through the suppression of cytokine and antimicrobial functions [42,43,44]. Researchers have found that itaconate also participates in the induction of immune tolerance and innate immune memory in human monocytes through immune-responsive gene 1 (IRG1) and succinate dehydrogenase polymorphisms [45]. Apart from citrate and itaconate, the contents of succinate, fumarate, and malate are accumulated in trained innate monocytes [39,45]. Succinate has been shown to regulate inflammatory responses during innate immune memory by activating the transcription factor HIF-1α, which is involved in angiogenesis [46] and the modulation of a series of genes, such as inflammatory mediator IL-1β [18,26]. Fumarate also contributes to innate immune memory by inhibiting the activity of KDM5 histone demethylases [31]. However, in contrast to the trained proinflammatory M1 macrophage, the process of the TCA cycle in M2 macrophages is still intact because uridine diphosphate N-acetylglucosamine is still synthesized from glutamine and glucose and is extensively used for glycosylation [18]. Therefore, according to the available research, it may be possible to approach the modulation of innate immune memory and the polarization of macrophages during inflammatory diseases via interventions in the TCA cycle.

### 3.3. Lipid Metabolism

The disruption of normal lipid metabolism in macrophages is a crucial induction factor for the formation of foamy macrophages and atherosclerosis. A growing body of research indicates that fatty acid metabolism participates in the creation of innate immune memory and might play a crucial role in the pathogenesis of atherosclerosis. In general, fatty acid synthesis is related to a proinflammatory macrophage phenotype due to the induction of ROS production, intracellular stress (e.g., ER), and the activation of NLRP3 inflammasomes [47]. The increased unsaturated fatty acids (i.e., arachidonic acid, oleic acid, and linoleic acid) can upregulate the production of IL-1α by uncoupling mitochondrial respiration and inducing a proinflammatory phenotype in atherosclerosis [48]. Endogenously derived triacylglycerides can be catabolized by β-oxidation to provide acetyl CoA for OXPHOS in mitochondria [49]. A recent study showed that the higher contents of polyunsaturated fatty acids in monocytes stimulated by Pam3Cys (TLR ligand) can activate peroxisome proliferator-activated receptor-gamma (PPAR-γ), which is closely associated with the establishment of innate immune memory during atherosclerosis [50]. In vivo assays subsequently demonstrated that a reduction in the metabolites involved in arachidonic acid and linoleic acid metabolism induced by β-glucan was associated with elevated innate immune responses and memory in hematopoietic stem and progenitor cells [51].

Cholesterol is an essential component of LDL and cellular membranes, and its excess accumulation in macrophages is the direct reason for the formation of foam cells during atherosclerosis. In the cytosol, acetyl CoA derived from citrate serves as an acetyl donor for histone acetylation and promotes fatty acid and cholesterol biosynthesis, which are primarily controlled by the enzyme 3-hydroxy-3-methylglutaryl CoA reductase (HMGCR). This process is important for the inflammatory functions of the innate immune cells because cholesterol can affect the inflammatory signaling transduction pathways through cellular membranes and can modify multiple proteins that are involved in inflammatory processes via prenylation [52]. The pharmacological repression of HMGCR by statins, an effective drug for attenuating atherosclerosis, can inhibit the innate immune memory induced by β-glucan and oxLDL [53]. Cholesterol synthesis pathways and metabolites are closely associated with the establishment of innate immune memory. An analysis of transcriptomes found that the synthesis process of cholesterol can be upregulated in trained macrophages induced by β-glucan [11]. Mevalonate, a metabolite of the cholesterol biosynthesis pathway, is involved in the regulation of innate immune memory, which can also be blocked by statins [53]. Studies indicate that the activation of the cholesterol synthesis pathway might be an essential process for the induction of innate immune memory, rather than the synthesis of cholesterol itself.

The nuclear liver X receptor (LXR) is a crucial transcription factor for the regulation of cholesterol and fatty acid metabolism genes. The activation of LXR can promote a proinflammatory trained immunity phenotype by increasing lactate and acetyl CoA production, upregulating sterol regulatory element-binding protein (SREBP) expression, and activating the mevalonate pathway and IL-1β signaling in human monocytes induced by Bacillus Calmette–Guérin (BCG) [54]. However, the functions of other important lipid metabolism regulators, such as SREBP and PPARs, in the establishment of innate immune memory remains to be explored.

### 3.4. Amino Acid Metabolism

Amino acids are essential molecules among various metabolism processes and provide necessary precursors via glutamine metabolism for the TCA cycle and fatty acid synthesis during the activation of innate immune memory, such as succinate and fumarate [31,55]. H3K27Ac is an important promoter and enhancer of histone H3 marks in macrophages’ innate immune memory [11]. Glutamate can be metabolized to α-ketoglutarate and finally to citrate in the TCA cycle. This contributes to the generation of cytosolic acetyl CoA, which serves as a histone acetyltransferase [56]. Moreover, acetyl CoA can be used in the fatty acid synthesis and cholesterol biosynthesis processes, which are important pathways that are upregulated in innate immune memory monocytes [11]. Glutamine metabolism can generate mitochondrial NADH through malate, and NADH can be transported to the cytoplasm for the production of NADPH, which participates in the cholesterol synthesis pathway. It has been reported that the inhibition of glutamine metabolism can decrease the production of proinflammatory cytokines in monocytes and impair the establishment of the innate immune memory induced by β-glucan [31]. In addition, in the process of atherosclerosis, the triglyceride content can be downregulated by glycine, alanine, leucine, and cysteine, while glutamine and glutamate can induce triglyceride accumulation in macrophages, which might be due to the participation of glutamine metabolism in fatty acid metabolism [57]. Further research on glutamine metabolism in the building of innate immune memory is needed, and a clearer understanding of this process might aid in our understanding of the mechanisms of atherosclerosis and aid in the development of therapies for this condition.

## 4. Innate Immune Memory Mediated Epigenetic Rewiring Is an Important Modulator in Atherosclerosis

The rapid and coordinated transcription of immune signaling molecules and cytokines supports a successful innate immune response. With innate immune memory, the epigenetic reprogramming of a large number of immune genes and their related cis-regulatory elements in innate immune cells occurs. For instance, macrophages can react more quickly or with a qualitatively different transcriptional response and polarize to a proinflammatory phenotype when challenged with endogenous or exogenous stimuli [22]. The molecular mechanisms underlying the altered responsiveness and transcriptional reprogramming of some inflammatory genes are well understood, but evidence supports the involvement of multiple regulators in this process, including chromatin reorganization, DNA methylation, histone modifications (such as acetylation, methylation, ubiquitination, phosphorylation, and recently lactylation), and the transcription of long noncoding RNAs (lncRNAs) [16,58]. The dysregulation of macrophage phenotypes is a primary driver of inflammatory diseases such as atherosclerosis, type 2 diabetes mellitus, and rheumatoid arthritis, especially in the case of macrophages with innate immune memory. In atherosclerosis, monocytes and macrophages are exposed to oxLDL, cholesterol crystals, inflammatory cytokines, and other inflammatory stimuli. All these factors induce not only a metabolic response but also extensive interactions, leading to epigenetic and transcriptional heterogeneity in macrophages in atherosclerotic lesions. Thus, understanding the epigenetic landscape of plaque macrophages might be a potential therapeutic approach to regulate proatherogenic phenotypes and suppress the formation of plaques. In this section, we review the various functions of epigenetic modifications in the development of atherosclerosis that have been reported most recently.

### 4.1. DNA Methylation

**Figure 3:**
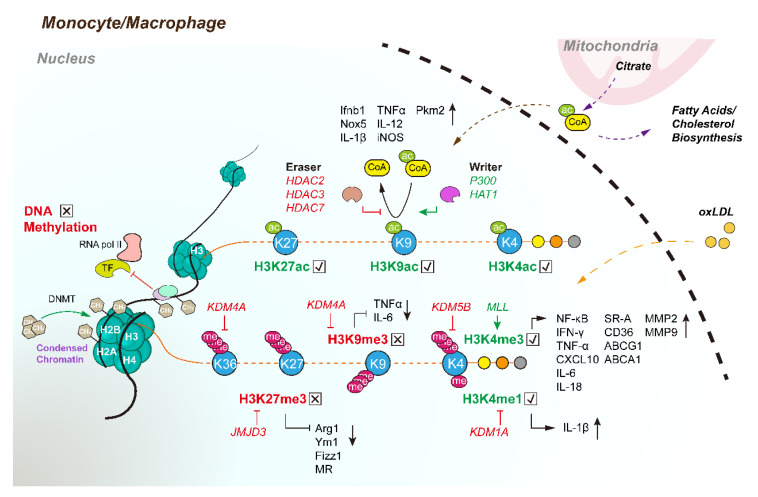
Epigenetic modifications of atherogenesis-related genes regulated by “Writer” and “Eraser” in monocytes/macrophages. DNA methylation can be regulated by DNA methyltransferase (DNMT), which increases the negative charge of DNA through increased CH_3_^−^, thereby leading to condensed chromatin. It can also attract proteins to specifically bind and modify DNA, indirectly repelling the access of transcription factors to DNA binding sites. Histone methylation can either trigger or suppress the transcription of target genes. Most atherogenesis-related genes are modified by H3 histone methylation and acetylation, which can be dually regulated by writers (e.g., MLL, p300, and HAT1) and erasers (e.g., KDM4A, KDM5B, KDM1A, JMJD3, and HDACs). Citrate can convert to acetyl CoA as an acetyl donor for histone acetylation and fatty acid and cholesterol biosynthesis. H3K4me1, H3K4 monomethylation; H3K4me3, H3K4 trimethylation; H3K4ac, H3K4 acetylation; MLL, mixed-lineage leukemia, HAT1, histone acetyltransferases, KDM, histone lysine demethylase; JMJD3, jumonji domain-containing protein 3, HDACs, histone deacetylases.

DNA methylation is an important biochemical process in which a methyl group is often added to a carbon atom of cytosine in cytosine-paired-with-guanine (CpG) dinucleotide sequences. It usually occurs at the gene promoter and enhancer elements and is a typical suppressive modification of gene transcription. Methylation can directly interfere with the binding of transcription factors and DNA, or it can attract proteins to specifically bind and modify DNA, indirectly repelling the access of transcription factors to DNA binding sites (Figure 3) [59]. When the binding sites are methylated, transcription factors, such as cyclic AMP response element binding protein (CREB), adaptor protein complex 2 (AP-2), and EBP-80, lose their affinity for genes [60,61,62]. DNA methylation is traditionally divided into de novo methylation and maintenance methylation, which are regulated by DNA methyltransferase (DNMT) [63]. DNA can undergo demethylation either passively or actively [64]. Passive DNA demethylation occurs when DNA replicates, while active DNA demethylation can be genome-wide through reproduction or can be locus-specific in somatic cells responding to certain signals [65].

DNA methylation abnormalities are associated with various diseases, such as autoimmune diseases, cancer, and atherosclerosis [66,67,68,69]. A marker of DNA methylation, 5-methylcytosine (5-mC), is higher in peripheral blood mononuclear cells (PBMCs) in coronary heart disease (CHD) and atherosclerosis patients [70]. In recent years, increasing numbers of atherosclerosis-related genes have been confirmed to undergo DNA methylation and modulate the progression of atherosclerosis. In support of the potential role of infection-induced innate immune memory in atherosclerosis, a number of genes associated with atherogenesis, including proatherogenic genes encoding chemokines and cytokines, and genes responsible for macrophage foamy cell formation and plaque instability have highly enriched histone H3K4me3 modifications in β-glucan-trained macrophages [9]. PPAR-γ plays an important role in the polarization and inflammatory cytokine production of macrophages [71,72]. Transgenic mice with a macrophage-specific overexpression of DNMT1 demonstrated upregulated DNA methylation in the PPAR-γ promoter, which led to the suppression of PPAR-γ-mediated anti-inflammatory effects and the development of atherosclerosis [73]. Dysferlin (DYSF) is an important molecule related to monocyte differentiation and inflammatory cytokine release [74,75], while the hypermethylation of DYSF’s promoter can upregulate its expression and facilitate the activation of monocytes, which further promotes the pathogenesis of atherosclerosis [76]. Homocysteine is considered to be an independent risk marker for atherosclerosis diagnosis and participates in the development of atherosclerosis. Researchers have found that increased homocysteine levels are related to increased *SMAD7* methylation levels. *SMAD7* is characterized by promoter hypermethylation and low expression in atherosclerotic plaques compared with normal arteries. Generally, DNA methylation exhibits the repressive function of gene transcription. Targeting aberrant DNA methylation might be one of the most important approaches in the prevention and cure of atherosclerosis.

### 4.2. Histone Modifications

Histone modifications primarily occur at lysine residues and include methylation, acetylation, ubiquitination, and phosphorylation, although other amino acids display complex modifications as well. However, the most common histone modifications are methylation and acetylation, and both of these are regulated by two reciprocal histone-modifying enzymes, which are named “writer” and “eraser” [77]. Interestingly, the innate immune memory of monocytes and macrophages can induce extensive epigenetic modifications, especially methylation and acetylation modifications at the histone level [6]. Meanwhile, these modifications are crucial regulators of inflammation- and metabolism-associated genes, which have close relationships with the development of atherosclerosis.

#### 4.2.1. Histone Methylation

Histone methylation can either trigger or suppress the transcription of target genes, depending on which amino acid residue is methylated and how many methyl groups are added [78]. Histone H3 is the primary site of histone methylation, which is generally catalyzed by lysine residues (Lys or K) in one of three states: mono-, di-, or trimethylated. Typical gene-activating methylated modifications include H3K4, H3K36, and H3K79, with H3K4 monomethylation (H3K4me1) marking enhancers (Table 1) [79], H3K4me3 modifying promoters [80,81], and H3K36 and H3K79 occurring mainly over gene bodies [82,83,84]. H3K9 and H3K27 methylations are generally gene suppression markers [82]. 

H3K4me3 is an important modification of macrophages during inflammation and inflammatory diseases that is regulated by mixed-lineage leukemia (MLL) and lysine demethylase 5B (KDM5B). MLL is involved in M1 polarization because MLL-mediated H3K4 methyltransferase activity and H3K4me3 levels are upregulated in M1 macrophages but decreased in M2 macrophages [85]. The H3K4me3 enrichment induced by MLL results in primed proinflammatory gene promoters, such as *NF-κB* [86], *IFN-γ*, *TNF-α* [87], and *CXCL10* [88,89]. Evidence has revealed the role of H3K4me3 in macrophages in chronic inflammatory diseases such as atherosclerosis. For instance, oxLDL-induced macrophages demonstrated high levels of proinflammatory genes (e.g., *IL-6*, *IL-18*, *TNF-α*, and *MCP-1*) and matrix metalloproteinase (*MMP2* and *MMP9*) due to the upregulation of promoter H3K4me3 modification via TLR signaling [9]. Moreover, H3K4me3 can also promote foam cell formation by increasing scavenger receptors (e.g., *SR-A* and *CD36*) and decreasing ATP-binding cassette transporters (such as *ABCG1* and *ABCA1*) when macrophages are exposed to oxLDL [9,90]. In contrast, the inhibition of H3K4me3 by lysine methyltransferase inhibitors could suppress oxLDL-induced proinflammatory gene expression in macrophages and foam cell formation [89]. A recent study revealed that the H3K4me1 of the IL-1β promoter could be increased by the tumor suppressor gene *SNF5* through the downregulation of KDM1A in macrophages, leading to enhanced IL-1β levels, thereby promoting the formation of atherosclerosis [91]. 

However, H3K9me3 and H3K27me3 are typical suppressive epigenetic modifications. Both have been proven to decrease in inflammatory cells within advanced plaques compared with early lesions and healthy carotid arteries [12]. Patients with familial hypercholesterolemia (FH) have an enhanced risk of atherosclerosis due to the innate immune memory mediated higher levels of proinflammatory and anti-inflammatory cytokines following TLR stimulation. A chromatin immunoprecipitation–qPCR analysis revealed downregulated H3K9me3 as well as significant enrichment of H3K4me3 at the promoter *TNFA* in FH patients [92]. The jumonji C-terminal domain-containing enzyme family is the primary group of KDMs. Importantly, jumonji domain-containing protein 3 (*JMJD3*) is involved in the inflammatory gene expression and polarization of macrophages by specifically catalyzing the demethylation of H3K27me3 [93,94]. In human abdominal aortic aneurysms and atherosclerotic plaques, *JMJD3* was upregulated by increased levels of inflammatory cytokines, and the repressive H3K27me3 level was decreased during monocyte–macrophage polarization [95]. The inhibition of *JMJD3* preserves H3K27me3 on inflammatory gene promoters and attenuates macrophage-mediated inflammation, which can be regulated by IFN-β via the JAK/STAT pathway [93]. Research has also revealed that *JMJD3* could be regulated by the proinflammatory serum amyloid A (SAA) and is essential for SAA-induced foamy macrophage formation when exposed to oxLDL [96]. In turn, SAA-induced proinflammatory gene expression was potently suppressed by *JMJD3* knockdown or an inactive mutation due to the restoration of the H3K27me3 level. Other studies also demonstrated that *JMJD3-*mediated H3K27me3 demethylation at the promoters of M2 genes promoted M2 polarization (e.g., *Arg1*, *Ym1*, *Fizz1,* and *MR*) via the IRF4 pathway but was dispensable for M1 polarization [97]. In addition, H3K9 and H3K36 modifications can be demethylated by histone lysine demethylase 4A (KDM4A, also known as JHDM3A and JMJD2A), another member of the JMJD family [98]. The knockdown of *KDM4A* suppresses the inflammatory cytokine expression induced by oxLDL and M1 polarization, along with enhanced Arg1 and VEGF expression [99]. Taken together, histone methylation modifications can be regulated by key writers and erasers, and these may be promising targets for the development of anti-inflammatory therapies for chronic-inflammation-induced atherosclerosis.

#### 4.2.2. Histone Acetylation

Histone acetylation generally occurs at lysine residues by neutralizing the positive charge, which may weaken the interactions between histones and DNA with a negative charge. Thus, acetylation usually induces gene expression by increasing the accessibility of transcription factors and RNA polymerase II [100]. It has been reported that histone acetylation is involved in the regulation of the inflammatory gene transcription of macrophages [101,102]. These histone acetylation and deacetylation modifications are regulated by histone acetyltransferases (HATs) and deacetylases (HDACs). Only a few HATs have been revealed to be involved in macrophage regulation, while many HDACs participate in the regulation of macrophage polarization and functions relevant to atherosclerosis. 

It has been confirmed that H3K9ac and H3K27ac are associated with enhancer and promoter activities [103]. When treated with LPS, THP-1 macrophages showed upregulated H3K9ac, H3K27ac, and H3K4me2, while this state was normalized by zerumbone through decreased HDAC3 [104]. Within human atherosclerotic plaques, H3K27ac, H3K9ac, and HATs (e.g., p300 and HAT1) are colocalized with NADPH oxidase 5 (Nox5), which contributes to oxidative stress in atherogenesis, macrophages, and lipid-rich deposits [105]. LPS enhances the levels of H3K27ac and H3K9ac and recruits p300 and HAT1 to the promoter of Nox5 in human macrophages, while the pharmacological inhibition of HATs potently suppresses the gene and protein levels of Nox5. TLR- and TNF-induced IFN-β-JAK-STAT loops are an important component of M1 activation. Interestingly, LPS-induced histone acetylation at the promoter of *Ifn-β* is significantly abolished in HDAC3-deficient macrophages [106]. A recent study demonstrated that class IIa HDACs (e.g., HDAC7) are key molecular links between TLR-induced aerobic glycolysis and macrophage inflammatory responses [107]. The proinflammatory and glycolytic enzyme Pkm2 directly interacts with the C-terminal deacetylase-containing domain of Hdac7, which leads to the deacetylation of cytoplasmic Pkm2 and the activation of proinflammatory responses, while silencing *HDAC7* in human macrophages results in reduced IL-1β release and LPS-induced CCL2. Similarly, the knockdown of HDAC2 contributes to a decrease in proinflammatory genes such as TNFα, IL-12, and iNOS following LPS stimulation via abolishing the direct binding to their promoters in macrophages. Moreover, it has been reported that the expression of *ABCA1* can be enhanced by miR-452-3p or miR-328-5p through inhibiting the deacetylation function of HDAC3 in THP-1 macrophages, thereby preventing atherosclerosis [108]. During M2 polarization, H3K27ac and H3K4me1 in *Arg1* and *Mrc1* can be enhanced by mediator 1 (MED1), which plays a suppressive role in the development of atherosclerosis [109].

Many studies have attempted to unravel the significance of epigenetic modifications in monocytes and macrophages as crucial causes and results of the progress of atherosclerosis. Thus, research has been performed on how and when DNA and histone modifications control the expression of metabolic and inflammatory genes, thereby repressing or promoting the development of atherogenic lesions. Monocytes and macrophages exhibiting innate immune memory demonstrate extensive epigenetic rewiring, regulating the inflammatory responses and the formation of lesions. Thus, it seems that epigenetic rewiring is the basic and crucial induction mechanism for the development of atherosclerosis.

**Table 1:** Histone modifications and their functions in monocytes and macrophages during atherogenesis.

Modification	Function in Transcription	Writer	Eraser	Functions in Atherosclerosis
H3K4me1	Positive	KMT2	KDM1A	Promotes IL-1β expression [91]
H3K4me3	Positive	MLL	KDM5B	Activates proinflammatory genes, matrix metalloproteinase, and ABC transporters [85,86,87,88,89]
H3K9me3	Negative	KMT1	KDM4A, KDM3	Enriched at the promoter and suppresses TNFα and IL-6 [12,92]
H3K27me3	Negative	KMT6	JMJD3, KDM4A	Represses inflammatory genes (IL-6 and TNFα) and suppresses M1 polarization [12,93,94,95]
H3K9ac/H3K27ac	Positive	p300, HAT1	HDAC7, HDAC2	Enhances Nox5 in macrophages and activates M2 anti-inflammatory genes [109]

## 5. Endogenous and Exogenous Atherogenic Stimulations Build Innate Immune Memory in Monocytes and Macrophages

Chronic arterial inflammation in myeloid-derived monocytes, especially macrophages, plays a crucial role in atherosclerosis. Any factors that promote this inflammation can potently enhance the formation, erosion, and rupture of plaques in the development of atherosclerosis [24]. Meanwhile, the fact that the activated phenotype of monocytes and macrophages persistently leads to an enhanced proinflammatory response is a mark of innate immune memory. Therefore, much evidence indicates that endogenous stimulation, including oxLDL [37], aldosterone [10], and other exogenous factors, participates in the induction of innate immune memory (Figure 2). Innate immune cells treated with these atherogenic stimuli can be trained to develop a chronic proinflammatory and proatherogenic phenotype, resulting in the development of atherosclerosis [12]. In this regard, it is reasoned that innate immune memory can act as an important factor in inflammatory atherosclerosis. Thus, in this section, the endogenous and exogenous atherogenic stimulations involved in the establishment of innate immune memory in monocytes and macrophages are summarized.

### 5.1. oxLDL

OxLDL is one crucial mediator of atherosclerosis that triggers inflammatory cascades and the formation of foam cells. It has been reported that oxLDL (but not LDL) can induce the establishment of innate immune memory via the epigenetic reprogramming of monocytes in vitro [9]. When briefly exposed to a low level of oxLDL in vitro, monocytes are programmed toward a proatherogenic state. The trained monocytes induce an atherogenic chemokine and cytokine response, including monocyte chemoattractant protein 1 (MCP-1), IL-8, IL-6, and TNF-α, on the secondary stimulation of oxLDL (Table 2). Foam cell generation and matrix metalloproteinases also increase in trained monocyte-derived macrophages, which might be due to the dyshomeostasis of cholesterol metabolism and the enhanced H3K4me3 at the promoter sites of proatherogenic genes. During this process, both *CD36* and *SR-A* are potently upregulated, while *ABCA1* and *ABCG1* are strongly downregulated, indicating the importance of the cholesterol metabolic pathway. In addition, oxLDL-induced innate immune memory might be dependent on TLR4 and -2 activation because the training effects can be partially reduced by their antagonists [9].

The glucose metabolism process can be rewired in oxLDL-induced innate immune memory in human monocytes. When human macrophages were trained with oxLDL, the glycolytic pathway enzyme associated genes, including *PFKP*, *PKM1*, and *PKM2*, tended to be upregulated following LPS stimulation, and the cells exhibited more proinflammatory cytokine production, including TNF-α and IL-6 [13]. In line with the “memory-like” reprogramming of cells, oxLDL can activate the mTOR/HIF-1α axis to induce the production of ROS throughout the monocyte-to-macrophage differentiation process, which is critical for oxLDL-induced innate immune memory. The pharmacological inhibition of the mTOR pathway results in a decrease in glycolysis and proinflammatory phenotypes in macrophages [37]. A recent study also revealed that the oxLDL-induced innate immune memory in macrophages was dependent on mitochondrial metabolic reprogramming. When macrophages were exposed to oxLDL for 24 h, TCA metabolites were demonstrated to be enriched, according to an omics profiling analysis. The OXPHOS activity was increased and may play an important role in the enhanced cytokine hyper-responsiveness of oxLDL-trained macrophages. The pharmacological blockade of the participation of glutamine and fatty acids in the TCA cycle could prevent oxLDL-triggered innate immune memory [38]. Furthermore, oxLDL-triggered innate immune memory can lead to decreases in extracellular progesterone levels following LPS stimulation when monocytes are pretrained with progesterone. Pharmacologic inhibition assays demonstrated that progesterone attenuates TNFα production in oxLDL-trained macrophages through the nuclear mineralocorticoid and glucocorticoid receptors [110]. As one of the crucial factors in the induction of atherosclerosis, oxLDL has demonstrated wide-ranging effects on metabolic reprogramming in trained macrophages. Thus, possible approaches that repress oxLDL-induced innate immune memory might provide an alternative pathway for the development of drugs for atherosclerosis.

### 5.2. Aldosterone

Aldosterone is a hormone that modulates electrolyte homeostasis and blood pressure. It is also one factor that directly contributes to the development of atherosclerosis, which has been proven in animal models [115]. A high aldosterone content shows proatherogenic effects, while the repression of the aldosterone pathway attenuates the adverse effects on cardiovascular health [116]. A recent study reported that a brief exposure to aldosterone could induce a proinflammatory feature in trained human macrophages by producing more TNFα and IL-6 in response to Pam3Cys restimulation through the activation of mineralocorticoid receptors [10]. Thus, aldosterone-induced innate immune memory provides a novel potential relationship between primary hyperaldosteronism and inflammatory atherosclerosis. The glycolysis process has been fueled and stabilized as the metabolic foundation in β-glucan-induced innate immune memory [25,31]; however, the glycolysis (by extracellular acidification) and OXPHOS (by oxygen consumption) metabolic processes did not seem to be affected when induced by aldosterone. Moreover, no differences were observed in the expression of oxLDL uptake transporter genes (*CD36* and *SR-A*), cholesterol efflux transporter genes (*ABCA1* and *ABCG1*), or foam cell formation in aldosterone-trained macrophages. In contrast, fatty acid synthesis (*ACACA* and *FASN*), fatty acid elongation (*ELOVL6*), the formation of long-chain fatty acids (*HACD4*), and the rate-limiting enzyme in the formation of monounsaturated fatty acids were all upregulated. Importantly, the inhibition of fatty acid synthesis inhibited the aldosterone-mediated innate immune memory, suggesting a fundamental role of fatty acid synthesis in aldosterone-induced innate immune memory [10].

In addition, aldosterone has been reported to promote macrophage lipid accumulation and the degradation of a fibrous cap that could drive the progression of atherosclerosis [116]. As one important determinant of innate immune memory, epigenetic modifications also occur in aldosterone-trained macrophages. An epigenetic analysis showed that H3K4me3 was observed to be consistently increased in the proximal promoters of both inflammatory genes (*TNF* and *IL-6*) and fatty acid biosynthesis genes (*ACACA*, *FASN*, and *ELOVL6*) [10]. Given that aldosterone can induce the innate immune memory of macrophages in vitro and its close association with advanced cardiovascular risk in humans, it is tempting to investigate aldosterone-mediated innate immune memory in vivo and its further functions in atherogenesis.

### 5.3. Cholesterol

Family hypercholesterolemia (FH), characterized by high levels of cholesterol, is closely associated with an increased risk of atherosclerosis. RNA-seq revealed that metabolic pathways (e.g., glycolysis, oxidative phosphorylation, and amino acid synthesis) and inflammatory pathways (e.g., the TNF and NF-κB signaling pathways) that facilitate the establishment of innate immune memory were enriched in FH patients [92]. This may partially be the result of increased H3K4me3 and decreased H3K9me3 in the promoter region of *TNFα*. However, in contrast to the inhibition function of statins in innate immune memory in vitro [53], treatment with statins does not revert the innate immune memory in vivo in FH patients [92]. In vivo studies showed that the high levels of cholesterol from Western diet (WD)-induced innate immune memory are also involved in the development of atherosclerosis because the contributions of immune cells in plaques and the potential anti-inflammatory capacity during plaque regression were attenuated. When mice were fed a WD for 4 weeks, the monocytic immune cells isolated from their spleens had potent enhanced TLR responses, suggesting a primed cell state. Interestingly, although the systemic cytokines returned to normal levels upon shifting mice back to a normal diet after the WD, the TLR responses of monocytes isolated from WD-to-normal-diet mice remained primed compared to cells isolated from mice fed with a single normal diet [111]. Histone demethylase Tet2 is a risk factor for atherosclerosis related to dysfunctional myelopoiesis and the activation of the NLRP3 inflammasome pathway [117]. Tet2 was found to be epigenetically triggered in myeloid progenitors in high cholesterol diet fed LDLR^-/-^ mice [111], which indicates that high cholesterol WD induced innate immune memory participates in atherogenesis.

### 5.4. β-Glucan

β-glucan can trigger long-term metabolic reprogramming in macrophages, contributing to an increased proinflammatory phenotype. The priming of healthy monocytes with β-glucan for 24 h demonstrated increased TNFα and IL-6 production upon restimulation with Pam3Cys after 6 days [7]. These enhanced proinflammatory cytokines in monocytes trained with β-glucan were associated with stable changes in histone trimethylation at H3K4 through a dectin-1/Raf-1 pathway [112]. The transcriptome and metabolome results demonstrated that several metabolic pathways, including the cholesterol synthesis pathway, glucose metabolism, and the glutaminolysis process, were upregulated in β-glucan-trained macrophages [25,31]. Monocytes trained with β-glucan exhibited decreased baseline oxygen consumption compared to naïve cells, suggesting a shift from oxidative metabolism toward glycolysis. β-glucan-induced innate immune memory in macrophages might exhibit a proatherogenic phenotype because cholesterol repression by statins was also observed to inhibit enhanced cytokine production and epigenetic rewiring, which are induced by both β-glucan and oxLDL [53]. It has been shown that the shift from OXPHOS to glycolysis in β-glucan-induced innate immune memory is dependent on the AKT/mTOR/HIF-1α pathway [11,25]. The inhibition of mTOR signaling with rapamycin resulted in a dose-dependent inhibition of the innate immune memory induced by β-glucan. Moreover, the epigenetic signals at the promoters of genes in the mTOR pathways were potently enhanced in β-glucan-trained monocytes compared with cells cultured with medium. Based on the identification of glycolysis as a fundamental pathway in β-glucan-induced innate immune memory, an important regulatory role for metabolic reprogramming in innate immune cells is highlighted. Additionally, this provides a potential therapeutic target for the treatment of both inflammatory and infectious diseases.

### 5.5. Mevalonate

The metabolite of the cholesterol synthesis pathway, mevalonate, is one of the regulators of innate immune memory through the activation of mTOR and the IGF1 receptor (IGF1-R) and the subsequent induction of histone modifications in inflammatory pathways [53]. Human and mouse monocytes exposed to exogenous mevalonate can recapture oxLDL and β-glucan-induced trained phenotypes. It has also been demonstrated that monocytes pretreated with mevalonate show augmented cytokine production upon restimulation with LPS. However, this increased cytokine production can be repressed by a nonselective methyltransferase inhibitor. Mechanically, mevalonate induces the enrichment of H3K4me3 in the promoters of *TNFα* and *IL-6*, which is similar to the innate immune memory triggered by oxLDL and β-glucan [9,112]. In addition, more lactate production can be observed in mevalonate-trained macrophages, indicating that the activation of the glycolytic pathway is similar to the effect of β-glucan-trained macrophages [25]. The repression of glycolysis by 2-deoxyglucose or the mTOR pathway with rapamycin inhibits mevalonate-induced innate immune memory. In previous studies, insulin-like growth factor 1 receptors (IGF1-R) were found to be affected by mevalonate [118]. Bekkering et al. confirmed that when IGF1-R was blocked the enhanced H3K4me3 and increased phosphorylation of mTOR, S6K, and 4EBP1 were diminished, suggesting the epigenetic alteration of mevalonate via IGF1-R [53]. Thus, the discovery of the role of mevalonate in the induction of innate immune memory has important consequences for hyperinflammatory diseases. As one important intermediate metabolite in cholesterol synthesis, the inhibition of mevalonate-mediated pathways might be an effective therapy for atherosclerosis.

### 5.6. Lipoprotein(a)

Lipoprotein(a) (Lp(a)), a kind of LDL-like particle, is composed of apolipoprotein(a) (apo(a)) covalently bound to apolipoprotein B-100 (apoB). The atherogenic function of Lp(a) has already been confirmed by genome-wide association studies and meta-analyses of epidemiological studies, which demonstrated a relationship between Lp(a) and cardiovascular diseases (e.g., stoke, myocardial infarction, and calcific aortic valve stenosis) [113,119]. Consistent with the known atherogenic mechanisms of oxLDL, the atherogenic capacity of Lp(a) might be dependent on the interaction of apoB with the accessory molecules within the arterial wall that promote retention [120]. Lp(a) is also the primary carrier of proinflammatory oxidized phospholipids (OxPL) and an important molecule that induces innate immune memory. Healthy monocytes incubated with the plasma of patients with elevated Lp(a) for 24 h showed increased IL-6 and TNFα production upon ex vivo restimulation with Pam3Cys. However, this innate immune memory of monocytes can be repressed by anti-OxPL antibodies, suggesting a regulatory correlation with OxPL, while monocytes isolated from subjects with high Lp(a) acquired an enhanced capacity to produce proinflammatory cytokines upon stimulation in vitro and remained in a long-lasting primed state [7]. In contrast, the potent lowering of Lp(a) levels caused by apoA antisense inhibition can reverse the proinflammatory phenotype of circulating monocytes in patients with cardiovascular diseases [114].

## 6. Conclusions and Perspective for Therapies

A large and growing body of evidence supports the idea that the exposure of the innate immune system to a variety of endogenous or exogenous atherogenic stimulations could induce the establishment of innate immune memory. Trained monocytes and macrophages enhance the production of proinflammatory chemokines/cytokines and promote the formation of foam cells. These trained cells exhibit potent atherogenic phenotypes. During the establishment of innate immune memory, metabolic and inflammatory pathways are modulated, involving the rewiring of glycolysis, OXPHOS, and fatty acid synthesis as well as epigenetic reprogramming at the level of histone modification. According to these theoretical foundations, statins and metformin were discovered and have been used for decades in clinical cases to treat atherosclerosis by interfering with key metabolic processes driven by innate immune responses. In contrast to the trained phenotype, macrophages can also develop a tolerant phenotype after sequential and repeated exposure to high-dose stimuli, such as LPS, endotoxin, and TNF [121,122,123]. In this context, tolerant macrophages tend to decrease proinflammatory cytokine expression but enhance anti-inflammatory genes through changing chromatin accessibility. Although limited studies revealed the relationship between macrophage tolerance and atherosclerosis, the reprogramming of tolerant macrophages plays a role in inducing immunosuppression in cancer and sepsis [124,125]. In this regard, more studies concerning trained and tolerant macrophages in atherosclerosis still need be conducted in the future.

Atherosclerosis is a complex inflammatory disease involving the alteration of various metabolic processes. More specific compounds, especially those that repress cholesterol synthesis and glycolytic processes according to the mechanism of innate immune memory, might have the potential to limit atherosclerosis. Thus, findings regarding metabolic reprogramming and epigenetic rewiring during innate immune memory in monocytes and macrophages might be helpful for the development of more targeted therapeutic approaches to prevent or treat atherosclerosis.

## Figures and Tables

**Table 2:** The metabolic and epigenetic reprograming in monocytes and macrophages induced by atherosclerosis-associated stimuli.

Atherosclerosis-Associated Stimuli	Metabolic Reprograming	Epigenetic Reprograming	Signaling Pathways
oxLDL [9,13,110]	Glycolysis, OXPHOS, TCA, cholesterol metabolism,	H3K4me3	TLRs-mediated inflammatory response, mTOR/HIF-1α axis
Aldosterone [10]	Fatty acid biosynthesis	H3K4me3	TLR2-mediated IL-6 and TNFα pathway, ROS production, fatty acid synthesis pathway
Cholesterol [92,111]	OXPHOS, glycolysis, amino acid synthesis	H3K4me3, H3K9me3	IFN signaling pathway, IL-1 and NLRP3 pathway
β-glucan [31,112]	Cholesterol synthesis, glycolysis, glutaminolysis	H3K4me3, H3K27me3, H3K27Ac	Dectin-1/Raf-1 pathway, AKT/mTOR/HIF-1α pathway, TLR2-mediated IL-6 and TNFα pathway
Mevalonate [53]	Cholesterol synthesis, glycolysis, TCA	H3K4me3, H3K27Ac	IGF1-R-mTOR signaling pathway
Lipoprotein(a) [25,113,114]	-	-	TLR-mediated inflammatory response

## Data Availability

Not applicable.
